# Influence of Exercise Order on Electromyographic Activity During Upper Body Resistance Training

**DOI:** 10.2478/hukin-2014-0127

**Published:** 2014-12-30

**Authors:** Rafael Soncin, Juliana Pennone, Thiago M. Guimarães, Bruno Mezêncio, Alberto C. Amadio, Júlio C. Serrão

**Affiliations:** 1 Laboratory of Biomechanics; School of Physical Education and Sport; University of São Paulo (USP); São Paulo; Brazil.

**Keywords:** neuromuscular activation, exercise order, strength training

## Abstract

The purpose of this study was to investigate the effects of exercise order on electromyographic activity in different muscle groups among youth men with experience in strength training. Three sets of 8 RM were performed of each exercise in two sequences order: (a) sequence A: bench press, chest fly, shoulder press, shoulder abduction, close grip bench press and lying triceps extension; (b) sequence B: the opposite order. The electromyographic activity was analyzed in the sternocostal head of the pectoralis major, anterior deltoid, and long head triceps brachii, normalized for maximal voluntary isometric contraction. The muscles activity of the sternocostal head of the pectoralis major, anterior deltoid, and long head triceps brachii showed significant interaction between sequence and exercise. The sternocostal head of the pectoralis major showed considerably higher activity in sequence A (100.13 ± 13.56%) than sequence B (81.47 ± 13.09%) for the chest fly. The anterior deltoid showed significantly higher electromyographic activity in sequence B (86.81 ± 40.43%) than sequence A (66.15 ± 22.02%) for the chest fly, whereas for the lying triceps extension, the electromyographic activity was significantly higher in sequence A (53.89 ± 27.09%) than sequence B (34.32 ± 23.70%). For the long head triceps brachii, only the shoulder press showed differences between sequences (A = 52.43 ± 14.64 vs. B = 38.53 ± 16.26). The present study showed that the exercise order could modify the training results even though there was no alteration in volume and intensity of the exercise. These changes may result in different training adaptations.

## Introduction

Resistance training results in health benefits such as improved performance in sports as well as helping in prevention and injury treatment. This is achieved through adaptation processes that are expressed by an increase in strength, power, hypertrophy and muscular endurance. In order to induce these adaptations, the variables involved in strength training prescription, such as exercise type, intensity, volume, frequency, rest intervals between sets and exercise order should be comprehended (ACSM, 2009).

As for the exercise order, strength training sessions are recommended to start with multi-joint exercises involving large muscle groups with more complex neuromuscular control, and end with single-joint exercises involving small muscle groups and with lower control demand of the central nervous system (ACSM, 2009; Fleck and Kraemer, 1987; Sforzo and Touey, 1996; Wathen, 1994; Zatsiorsky, 1995). These recommendations are largely based on empirical evidence, nonetheless little is reported about exercise order in comparison to the volume/intensity studies (Simão et al., 2012b).

Simão et al. (2010) verified the chronic effect of exercise order in non-trained men, where a significant increase in 1 RM for the exercises performed at the beginning of the session was found for both cases of exercise order, i.e. multiple to single-joint exercises or vice-versa. Several studies that examined acute responses resulting from alterations to exercise order have shown the same conclusion. It was found that fewer repetitions were executed in the exercises performed at the end of the strength training session. These results were independent whether the exercises were multi-or single-joint, or involved large or small muscle groups (Farinatti et al., 2013; Romano et al., 2013; Simão et al., 2005, 2007, 2012a, 2012b). This evidence suggests that the exercise executed at the beginning of the session had a greater strength gain, regardless of its being a multi or single joint exercise, for a large or small muscle group.

A tool that may contribute to better understanding of the effects of exercise order on strength training is the electromyographic activity (EMG). This method allows to verify whether the pattern of activation in different exercises is altered when they are at the beginning or at the end of the training session (Butler et al., 2013; Helgadottir et al., 2011; Hubley-Kozey et al., 2002, 2014; Jamison et al., 2013; Qi et al., 2012; Sotiropoulos et al., 2010; Van Damme et al., 2013). Thus, the aim of this study was to analyze changes in the pattern of muscle activation between chosen exercises applied in different order.

## Material and Methods

### Participants

Ten men (age, 26.7 ± 3.8 years old; body mass, 83.8 ± 5.3 kg; body height, 177.0 ± 0.4 cm) with at least 5 years of resistance training experience and without orthopedic injury history participated in this study. All of the subjects were used to training in a range of 8 to 12 RMs, performing the bench press, chest fly, shoulder press, shoulder abduction, close grip bench press and lying triceps extension exercises in their training routines. The experimental procedure was approved by the research ethics committee of the University of São Paulo and all of the subjects signed a consent form.

### Measures

Electromyography data were recorded from the sternocostal head of the pectoralis major, anterior deltoids and long head of triceps brachii muscles to investigate the effect of two different sequences of exercise, assessed on a minimum of 4 non-consecutive days.

The first two days were spent to test and to re-test 8 repetitions maximum (8RM) for all subjects; for all exercises, 48–72 hours prior to each session was the period of time in which the subject refrained from resistance training. The other two days were used for experimental sessions where the two different exercise sequences were performed separately for seven days using a counter-balanced cross-over design. The interval between the 8RM re-test and the experimental sessions was seven days. The two experimental sessions consisted of the same exercises under the same conditions performed, but in opposite exercise order.

The exercises used in each experimental session included the bench press, chest fly, shoulder press, shoulder abduction, close grip bench press and lying triceps extension. All of the exercises were executed with free weight. Sequence A (SEQ A) began with large-muscle group exercises and progressed towards small-muscle group exercises and multiple-joint exercises before single-joint exercises. Sequence B (SEQ B) used exact opposite order and began with small-muscle group exercises and progressed toward large-muscle group exercises and single-joint exercises before multiple-joint exercises. All of the exercises in both sequences were performed for 3 sets of 8RM with 2-min rest intervals of passive recovery (ACSM, 2009).

### Procedures

#### Determination of 8 Repetitions Maximum Load

The subjects performed the 8RM tests of each exercise. The strategies adopted to minimize errors in the 8RM tests were based on a previous study (Brennecke et al., 2009): (a) the subjects received standard instructions about data assessment and exercise performance techniques before testing, (b) the exercise technique execution was monitored and corrected when necessary, and (c) the subjects received a verbal encouragement during the test. The warm-up consisted of 8 repetitions with 50% of the usual training load for each exercise. During the 8RM tests, each subject had a maximum of 3 attempts at each exercise with a 5-min rest interval between attempts. If the subject did not accomplish 8RM in the first attempt, the load was adjusted by 1 to 2 kg before the next attempt. After the 8RM, the load in a specific exercise was determined; an interval no shorter than 20 min was given to the subject before their 8RM determination of the following exercise. The execution cadence was determined as 1 by 1 s for the concentric/eccentric phase controlled by a metronome, no pause was allowed between exercise phases. For a successful repetition, the complete range of motion was required. The 8RM re-test session consisted of the same procedures as the 8RM test session. If the 8RM loads were different between the sessions, a new session would be performed, but this procedure was not necessary for any subject.

#### Maximal Voluntary Isometric Contraction Procedure

The standard adopted to normalize EMG data was the maximal voluntary isometric contraction (MVIC) (Burden, 2010). The MVICs were performed at the beginning of each experimental session in the following positions: the sternocostal head of the pectoralis major muscle in a supine position with the right shoulder horizontally abducted at 90 degrees; the anterior deltoid muscle standing with the right shoulder at 90 degrees of flexion and the long head of triceps brachii muscle in a supine position with the right shoulder and elbow at 90 degrees of flexion. Four tests were performed for each muscle, in which the first three were used as a warm-up. The first two contractions were 10 s sub-maximal contractions, the third was a maximal contraction of 5 s and the last, a MVIC for 10 s. The rest interval between the contractions of the same muscle was 1 min, and between the muscles, 2 min. In sequence A of the experimental session, the MVIC was performed in the following order: sternocostal head of the pectoralis major, anterior deltoid and long head of triceps brachii; in sequence B of the experimental session, the MVIC was performed in opposite order.

#### Exercise Sessions

Seven days after the 8RM re-test, subjects performed one of the exercise sequences in a counter-balanced cross-over design. Seven days after performing the first exercise sequence, the second sequence was performed. A group of five subjects performed sequence A first and another group of five subjects performed sequence B first. The sessions began with the MVIC procedure and after a 2-min rest interval, subjects performed the assigned exercise sequence. In this way, the subjects did not need a new warm-up procedure. Each exercise sequence consisted of 3 sets of 8RM for each exercise with 2-min rest intervals between sets and exercises. During the exercise sessions, subjects were instructed to perform all of the sets with the same reference of the range of motion and execution cadence used during the 8RM test. The subjects were verbally encouraged during all the sessions.

#### Data Acquisition

A Lynx-EMG 1000 System (Lynx Tech) was used for EMG data collection. Unit specifications included a differential input impedance of greater than 10 MΩ, sample frequency of 2000 Hz, a gain of 1000×, and a common mode rejection ratio of greater than 100 dB at 60 Hz.

Electromyography data of PM, AD and TB muscle were collected. The motor point was used as the reference for electrode placement. The electrodes were placed at 2 cm from the motor point location in the direction of the muscle insertion. Motor point localization was performed by the use of an electric pulse generator (OMNI PULSI-901, QUARK, Brazil) with 2 stimulating electrodes: a passive electrode was placed on the lumbar area and an active one was placed over the muscle belly. The generator emitted 1-ms pulse train as tetanizing frequency (20 to 80 Hz). The impedance between electrode pairs, using a 25-Hz signal through the electrodes, was less than 5.0 kV. The most excitable point was considered to be the motor point. The sites for electrode placement were prepared by shaving and abrading the skin with fine sandpaper and cleansed with 70% isopropyl alcohol. Electrodes were positioned with a center-to-center distance of 20 mm. A ground electrode was attached over a bone at the clavicle. All of these procedures were performed by the same investigator.

Electromyographic data were synchronized with a kinematic system to define the beginning and the end of each repetition. Kinematic data were recorded by a digital camera (Panasonic, model PV-GS50S, 60Hz) and analyzed by the Peak Motus 8.0 system (Peak Performance Technologies, Inc.). Reflexive landmarks were placed at the bar or dumbbell.

##### Data Analysis

The EMG signals were filtered with a fourth order Butterworth band-pass from 20–450 Hz and a notch filter with cut-off frequency of 60 Hz and harmonics.

In the MVIC, an interval of 4 s established between the fourth and the eighth seconds of contraction was used for analysis (Burden, 2010). The RMS value was calculated through a moving window of 200 ms in steps of 100 ms. The largest RMS value was designated as the reference EMG and used for its normalization.

For exercise analysis, the RMS of each repetition of each series was calculated based on kinematic data. The average RMS of each series from the RMS values of each repetition was also calculated. Then, the average RMS value for each exercise from the RMS values of each series was calculated. All signal processing was run with the software Matlab (version 2009b; Mathworks, USA).

### Statistical Analyses

Results are presented as means ± SD. The normality and homoscedasticity of data were tested using the Kolmogorov-Smirnov and Levene tests, respectively. To check the differences of the EMG activity, we used a factorial analysis of variance with repeated measures and two factors, where one factor was the sequence and the other factor was exercise. A Student-Newman-Keuls (SNK) post hoc test was used when necessary. The level of significance was set at p≤0.05 for all statistical procedures. All analyses were performed in the software SigmaStat (version3.5; Systat, USA).

## Results

The values of 8RMs were: bench press − 91.71 ± 12.08 kg; chest fly − 26.71 ± 4.19 kg; shoulder press − 48.00 ± 2.65 kg; shoulder abduction − 13.86 ± 2.04 kg; close grip bench press − 67.14 ± 13.90 kg and lying triceps extension − 43.14 ± 12.27 kg.

The RMS values are expressed as Mean ± SD percentage of MVIC for each sequence and exercise in Table 1. The sternocostal head of the pectoralis major muscle showed significant values for the main effect of sequences (ME SEQ). All muscles analyzed showed significant differences for the main effect of exercises (ME EX) and for the interactions between the factors.

**Figure 1:**
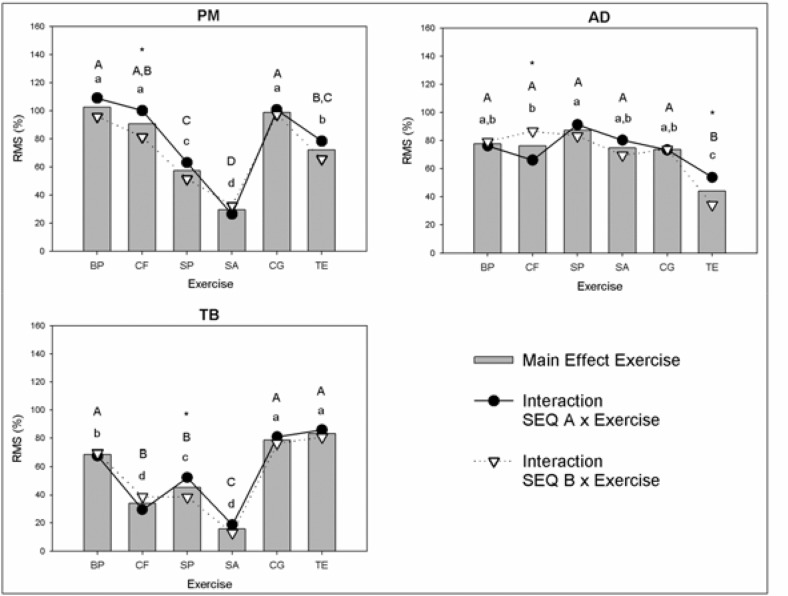
RMS of the EMG activity for PM, AD and TB in each exercise. PM = Sternocostal head of the pectoralis major; AD = Anterior deltoid; TB = Triceps brachii; BP = Bench press; CF = Chest fly; SP = Shoulder press; SA = Shoulder abduction; CG = Close grip bench press; TE = Lying triceps extension; ^a b c d^ SEQ A × Exercise Interaction Analysis; ^A B C D^ SEQ B × Exercise Interaction Analysis; Same letters representing no significant difference between exercises (p>0.05). * Exercise × Sequences Interaction Analysis (p<0.05).

For ME SEQ, the sternocostal head of the pectoralis major showed EMG activity significantly higher in sequence A (ES = 0.26; p<0.05). The results for ME EX are presented in Figure 1.

For the interaction Sequence × Exercise when the activities were compared with the same exercises among sequences, the chest flyes presented a significantly higher sternocostal head of the pectoralis major’s RMS in sequence A than sequence B (ES = 1.40) whereas the anterior deltoid in sequence B was significantly higher than sequence A (ES=0.63).

The lying triceps extension exercise showed the anterior deltoid’s RMS significantly higher in sequence A than sequence B (ES = 0.77). Furthermore, the shoulder press exercise showed a higher long head of triceps branchii’s RMS in sequence A than sequence B (ES = 0.90). The activity of all exercises within each sequence is presented in Figure 1.

## Discussion

The aim of this study was to analyze the electromyographic activity during two training sessions with different exercise order. The main findings were as follows: i) the exercise sequence can change the muscular recruitment; ii) the main cause of these changes in EMG signal seemed to be the muscle function in exercises and; iii) although the largest to smallest muscle sequence had an influence on neuromuscular activity, the multi-to-single-joint sequence seemed not to affect it.

TB showed significantly higher EMG activity during shoulder press in sequence A, probably because in sequence B the triceps exercises (close grip bench press and lying triceps extension) were executed earlier. Moreover, the primary muscle involved in the shoulder press is the anterior deltoid, thereby, when necessary, this muscle can make the exercise feasible even when the triceps are less active.

The anterior deltoid’s activity was significantly higher in sequence A than sequence B, and for both sequences its activity was lower than the other exercises. Muscle fatigue increases the variability of movements, changing the strategies of biomechanic coordination and / or a muscle activation pattern (Gates and Dingwell, 2010). Most likely, when the lying triceps extension was executed at the end of the sequence, it required a greater demand of stabilization due to the fatigue accumulated in the other exercises, and part of the stabilization was performed by the anterior deltoid.

In reference to the chest fly exercise, the anterior deltoid’s activation was significantly higher in sequence B whereas the sternocostal head of the pectoralis major had significantly higher activation in sequence A. One hypothesis for this increase in the RMS value is an alteration in the muscle recruitment pattern in function of previous executed exercises, where in the secondary agonist muscle, it increases its participation over the primary agonist.

It is known that different factors can change the recruitment pattern for the same task, such as low back pain (Butler et al., 2013; Helgadottir et al., 2011; Hubley-Kozey et al., 2002, 2014), changes in movement velocity (Van Damme et al., 2013), fatigue (Qi et al., 2012), anterior cruciate ligament injury (Jamison et al., 2013), and warming-up before training (Sotiropoulos et al., 2010). From our results, it seems that exercise order is another factor that can influence the pattern of muscular recruitment. Thus, the execution sequences of exercise influence not only the training volume (Farinatti et al., 2013; Romano et al., 2013; Simão et al., 2005, 2007, 2012a, 2012b; Spreuwenberg et al., 2006), but also the recruitment pattern, which may lead to different training adaptations.

It was expected that the muscles involved in intermediate exercises are less affected by the exercise sequence (Silva et al., 2009; Simão et al., 2005), however, it did not happen for the anterior deltoid in the present study. This probably occurred due to the primary or secondary agonist function played by the anterior deltoid in 5 of 6 analyzed exercises. Thus, the function of muscles in the exercises can be the principal factor that influences the sensitivity in exercise order.

The sternocostal head of the pectoralis major was the biggest muscle analyzed in this study and the only one which showed a difference in the main effect between the sequences and was found higher in sequence A. Therefore, the recommendation of the execution order from large to small muscles seems to affect the muscle activation.

However, muscle activity between multi and single-joint exercises (bench press and chest fly; shoulder press and shoulder abduction; close grip bench press and lying triceps extension) was the same when comparing sequences A and B. For example, the sternocostal head of the pectoralis major activity remained higher in the bench press when compared to the chest fly in both sequences. Thus, there is no electromyographic evidence that would justify the recommendation for the execution of exercises from multi to single-joint.

## Conclusion

The execution sequence of exercise affects neuromuscular activity during a training session. Thus, the sequence choice of exercises may influence training adaptations, even when there is no alteration in training volume. Nevertheless, initiating the session with large muscles showed an increase in recruitment of large muscles, and no evidence was found that the sequence multi to single-joint would influence muscle activity. Another factor that can affect muscle activity is the function performed by each muscle, regardless of its size or being single or multi-joint. Thus, based on the electromyographic activity, the exercises should be organized by grouping them according to the function of the involved muscles and their size.

## Figures and Tables

**Table 1:** RMS (%MVIC) of the EMG activity for PM, AD and TB.

	**PM #**			**AD #**			**TB #**		
	**SEQ A**	**SEQ B**	ME EX*	**SEQ A**	**SEQ B**	ME EX*	**SEQ A**	**SEQ B**	ME EX*
**BP**	109.04 ±12.30	95.94 ±21.28	102.49 ±18.16	76.30 ±18.83	79.36 ±19.28	77.83 ±18.56	67.81 ±16.38	69.74 ±20.08	68.78 ±17.81
**CF**	100.13 ±13.56	81.47 ±13.09	90.80 ±16.10	66.15 ±22.02	86.81 ±40.43	76.48 ±33.33	29.47 ±13.18	38.74 ±14.98	34.10 ±14.49
**SP**	63.18 ±22.87	51.61 ±33.56	57.39 ±28.49	91.31 ±11.73	83.30 ±10.26	87.31 ±11.46	52.43 ±14.64	38.53 ±16.26	45.48 ±16.63
**AS**	26.44 ±12.46	32.56 ±25.29	29.50 ±19.59	80.24 ±9.68	69.69 ±18.76	74.96 ±15.46	18.80 ±15.24	12.96 ±8.27	15.88 ±12.27
**CG**	100.07 ±26.80	97.38 ±24.53	98.72 ±24.96	73.42 ±18.09	73.96 ±14.99	73.69 ±16.12	80.91 ±13.31	76.32 ±21.64	78.62 ±17.58
**TE**	78.48 ±23.20	65.71 ±24.50	72.10 ±24.06	53.89 ±27.09	34.32 ±23.70	44.11 ±26.66	85.84 ±23.95	80.69 ±17.27	83.27 ±20.43
ME SEQ*	79.55 ±34.03	70.78 ±33.29	-	73.55 ±21.43	71.24 ±28.30	-	55.88 ±29.74	52.83 ±29.52	-

* p<0.05 for main effect;

# p<0.05 for interaction. RMS (%MVIC) of the EMG activity for PM, AD and TB. Values expressed as mean ± standard derivation. ME SEQ = Main effect for sequence; ME EX = Main effect for exercise; SEQ A = Sequence A; SEQ B = Sequence B; PM = Sternocostal head of the pectoralis major; AD = Anterior deltoid; TB = Triceps brachii; BP = Bench press; CF = Chest fly; SP = Shoulder press; SA = Shoulder abduction; CG = Close grip bench press; TE = Lying triceps extension
